# The Use of Artificial Intelligence Algorithms in the Diagnosis of Urinary Tract Infections—A Literature Review

**DOI:** 10.3390/jcm11102734

**Published:** 2022-05-12

**Authors:** Natalia Goździkiewicz, Danuta Zwolińska, Dorota Polak-Jonkisz

**Affiliations:** 1 Department of Pediatric Nephrology, University Hospital in Wroclaw, 50-556 Wrocław, Poland; 2 Department of Pediatric Nephrology, Wroclaw Medical Univeristy, 50-556 Wrocław, Poland; danuta.zwolinska@umed.wroc.pl (D.Z.); dorota.polak-jonkisz@umed.wroc.pl (D.P.-J.)

**Keywords:** urinary tract infections, artificial intelligence, machine learning, medical decision support system

## Abstract

Urinary tract infections (UTIs) are among the most common infections occurring across all age groups. UTIs are a well-known cause of acute morbidity and chronic medical conditions. The current diagnostic methods of UTIs remain sub-optimal. The development of better diagnostic tools for UTIs is essential for improving treatment and reducing morbidity. Artificial intelligence (AI) is defined as the science of computers where they have the ability to perform tasks commonly associated with intelligent beings. The objective of this study was to analyze current views regarding attempts to apply artificial intelligence techniques in everyday practice, as well as find promising methods to diagnose urinary tract infections in the most efficient ways. We included six research works comparing various AI models to predict UTI. The literature examined here confirms the relevance of AI models in UTI diagnosis, while it has not yet been established which model is preferable for infection prediction in adult patients. AI models achieve a high performance in retrospective studies, but further studies are required.

## 1. Introduction

Urinary tract infections (UTIs) are among the most common bacterial infections, affecting 150 million people each year [1]. UTI is a collective term describing infections that involve the colonization of pathogens found anywhere in the urinary system, comprising cystitis, pyelonephritis, renal abscess, urethritis, and prostatitis. In clinical practice, UTIs are categorized as uncomplicated or complicated. Uncomplicated UTIs include acute uncomplicated cystitis (AUC)—infection of the bladder or lower urinary tract—and acute uncomplicated pyelonephritis (AUP)—infection of the kidney or upper urinary tract—which occur in patients who have a regular, unobstructed urinary tract with no history of recent instrumentation. Complicated urinary tract infections can arise in a urinary tract that has metabolic, functional, or structural abnormalities. Complex UTIs may involve any part of the urinary tract. The main consequence of UTIs is that they critically increase the possibility of a failure of therapy.

UTIs are caused by both Gram-negative and Gram-positive bacteria, as well as by certain types of fungi. The dominant infectious microorganism for both uncomplicated and complicated UTIs is uropathogenic *Escherichia coli* (UPEC), although the distribution of pathogens causing UTIs varies [2].

The diagnostic process of a UTI is usually carried out based on a combination of clinical symptoms of infection and a positive urine analysis or culture [3]. Some of these features have been combined into clinical predictors, but the predictive values remain sub-optimal. Although the culture of the urine remains the gold standard for diagnosing and treating UTIs, technical considerations including the methods of collection of the urine as well as the time necessary for obtaining culture results remain problematic. Urine culture examination has the disadvantage of taking at least 48 h to produce an outcome. Rapid, cost-effective methods for UTI diagnosis are required as an alternative form of scanning. Moreover, the diagnosis of UTIs using clinical criteria alone has an error rate of approximately 33% [4]. Therefore, the development of better diagnostic tools for UTIs is essential for improving antimicrobial stewardship and to reduce the morbidity associated with this condition.

The choice of management options for UTIs depends on whether they are uncomplicated or complicated. Most guidelines for non-complex UTIs recommend treatment with empirical antibiotics; however, this accounts for a considerable percentage of antibiotic prescriptions. Anti-microbial drugs should not be prescribed excessively, as they may result in antibiotic overuse and contribute to the development of antimicrobial resistance. In the management of pyelonephritis, clinicians need to correctly differentiate between acute uncomplicated forms and complicated, often obstructive, forms of UTIs that require early appropriate imaging. Quick and proper treatment can prevent urosepsis.

As UTIs are a major issue in all age groups and are thus significant in clinical practice, a high level of diagnostic accuracy is crucial.

### Digitalization in Medical Field

Making sense of human language has been a goal of artificial intelligence researchers since the 1950s. Technological development in the health industry has increased significantly over the last 10–15 years. In most industrialized countries, a shortage of medical professionals has stimulated the need for technology, especially new and inventive implementations of artificial intelligence models and algorithms. Applying this kind of software in order to solve medical problems can prove to be highly beneficial, especially in terms of cutting costs, lowering the amount of required time and the need for human knowledge and resources, and reducing the number of medical errors.

Artificial intelligence, as an advanced science technology, has been widely used in medical fields to promote medical development, mainly considering the early detection [5], diagnosis [6], and management of diseases [7]. For instance, Secinaro et al. [7] in their research extensively described the impact and potential use of AI in healthcare. They pointed out that AI helps in diagnostic accuracy and has the potential to analyze health data by comparing thousands of medical records, thus providing efficient management of health services and places of care.

AI models can be used not only to identify UTIs, but also to recognize patients at highest risk for serious complications such as sepsis. The systematic review presented by Choudhury, A., and Asan, O. [5] indicates that AI-enabled decision support systems, when implemented correctly, can aid in enhancing patient safety by improving error detection, patient stratification, and drug management.

The objective of this study was to analyze current views regarding attempts at applying AI techniques in clinical practice, as well as to find promising methods to diagnose UTIs in more efficient ways. We also compared the currently used AI models and identified the most effective one.

## 2. Methods

Our narrative review contains a critical and objective analysis of the current knowledge on the use of AI in UTI diagnostics. This study was reported according to the Preferred Reporting Items for Systematic Reviews and Meta-Analysis (PRISMA) guidelines. We followed the PRISMA Checklist. Our protocol was registered with the Open Science Framework on 3 April 2022.

We searched for publications in the Pub Med, ProQuest, and Cochrane databases from January 2006 to August 2021.

The search strategy included randomized controlled trials, clinical trials, and observational studies. The reference lists of articles were examined for additional relevant studies. The keywords used in the search were initially determined by a preliminary review of the literature.

The final search query for PubMed was as follows: (“artificial intelligence” [MeSH] OR “artificial intelligence” OR “machine learning” [MeSH] OR “machine learning” OR “deep learning” [MeSH] OR “deep learning” OR “natural language processing” [MeSH] OR “natural language processing”) AND (“urinary tract infection*” [MeSH] OR “urinary tract infection*” OR “bacteriuria” [MeSH] OR ”bacteriuria”). The search was restricted only to English-language literature.

**Figure 1:**
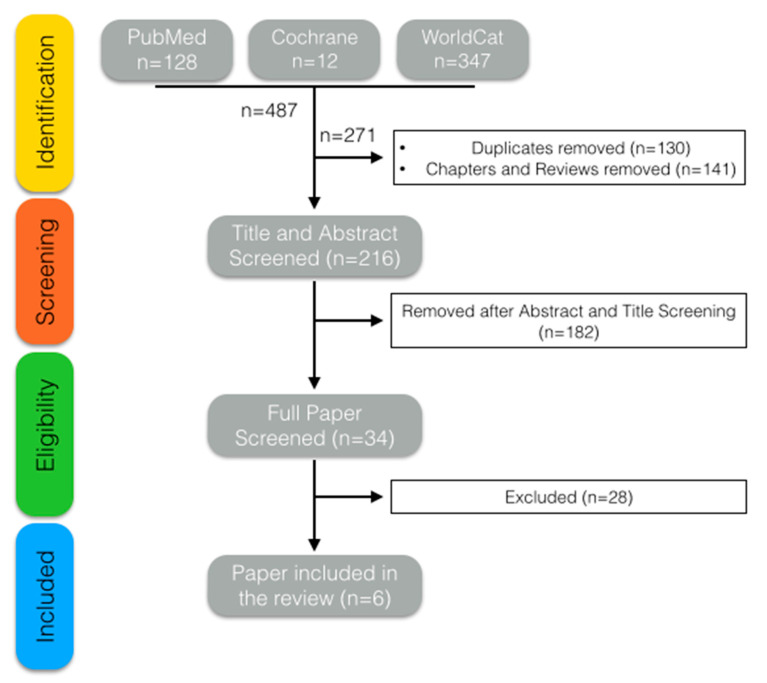
Search strategy.

We excluded any study if the data were insufficient for outcome assessment, when recurrent UTIs were analyzed, opinion/review papers, and studies involving the pediatric population. Six trials met the required criteria (Figure 1). The collected data from the chosen trials are summarized in Table 1.

## 3. Overview of AI Applications in UTI Diagnosis

AI is defined as the science and engineering of creating intelligent machines that behave in a way that could be considered intelligent if it was done by a human being [8]. One of the major branches of AI is machine learning, which is defined as the study of algorithms and statistical models that computer systems use to learn from sample data and past experience, without being explicitly programmed to perform specific tasks [9,10,11].

With the capacity to identify hidden patterns in the data, machine learning can be used to solve various problems, such as finding the associations between two variables, classifying subjects by certain criteria, making predictions based on baseline characteristics, and recognizing objects with similar patterns. Popular machine learning algorithms include support vector machine (SVM), random forest (RF), gradient boosting decision tree (GBDT), and artificial neural network (ANN) [12].

SVM is a well-known method, especially for classification where sample sizes are small. In a multidimensional environment, SVM is the linear separator between data samples that classify them by creating an optimal hyperplane.

RF is a technique that produces multiple classification and regression (CART) trees. Each tree is trained on a bootstrap sample of the original training data and searches a random subset of variables; thus, every tree is ”voting”. Classification is a result of the average vote of all of the trees.

ANN is a common method that consists of single or multiple layers, and it is made up of processing units that are called nodes/neurons. Signals travel though the network via nodes that are interconnected. There are three types of neurons: input (receives information), hidden (main task is extracting patterns), and output (responsible for final network result).

Boosting algorithms are becoming more and more popular because of their high interpretability, ease of implementation, and high prediction accuracy. There are several types of boosting algorithms, such as the AdaBoost algorithm, gradient boosting algorithm, and XG boost algorithm. Boosting algorithms produce a decision tree based on a sample of the training data. The main goal of the algorithm is to build a basic weak classifier, and then the algorithm uses it for continuous learning.

Taylor et al. [13] performed a single-center, multi-site, retrospective cohort analysis of 80,387 adults who visited the emergency department considering urine culture results and UTI manifestation. These authors tried to answer the question of which currently known AI algorithm has the highest specificity and sensitivity in UTI diagnosis using clinical symptoms, blood, and urine samples. They developed models for UTI prediction with six machine learning algorithms: RF, extreme gradient boosting, adaptive boosting, SVM, elastic net, and ANN using both laboratory and clinical data. Models were developed with both the full set of 211 factors and a reduced set of 10 variables (age, gender, UA nitrites, UA WBC, UA bacteria, UA blood, UA epithelial cells, history of UTI, and dysuria). UTI predictions were compared with the previous documentation of UTI diagnosis and antibiotic administration. Taylor RA et al. found that the top performing algorithm for both the full and reduced models was extreme gradient boosting (XGBoost), which had an area under the curve of 0.904 [8]. The XGBoost full and reduced models demonstrated greatly improved specificity in comparison with the provider judgment proxy of UTI diagnosis or antibiotic administration, while also demonstrating superior sensitivity when compared with the documentation of UTI diagnosis. The study concluded that the application of the algorithm in real life would allow approximately 1 in 4 patients to be re-categorized from false positive to true negative, and 1 in 11 patients to be re-categorized from false negative to true positive.

The literature suggests that approximately two-thirds of urine samples typically yield negative culture results [14]. Burton and colleagues [14] in their study tried to use artificial intelligence to reduce diagnostic workload without compromising the detection of UTIs. The researchers’ aim was to identify which markers in the urine samples were the most sensitive and specific in order to help diagnose UTIs without the need to culture. They retrospectively analyzed 212,554 urine reports. They used two methods of classification, a heuristic model and a machine learning approach, testing three algorithms (random forest, neural network, and extreme gradient boosting). The study concluded that, in a heuristic model, the combination of the white blood cell count and bacteria count showed the strongest correlation with the probability of significant bacterial growth on the culture. The optimum minimum thresholds for WBC and bacterial counts were found to be 30 μL and 100 μL, respectively. They found that, with the application of these criteria, there would be a 39.1% reduction in the number of samples needing culture and a sensitivity of 96% for the positive bacterial culture. For the machine learning algorithms, models were developed using the set of 16 factors. All of the machine learning algorithms outperformed the heuristic model. After further analysis, the authors concluded that the samples from pregnant patients and children (age 11 or younger) required independent evaluation [14]. It turned out that the best overall solution was to combine three extreme gradient boosting algorithms, trained independently for the classification of pregnant patients, children, and then all other patients. When combined, this system granted a relative workload reduction of 41% and a sensitivity of 95% for each of the stratified patient groups.

In their research, the Advanced Analytics Group of Pediatric Urology and ORC Personalized Medicine Group tried to create a model that could identify children with an initial UTI who were at the highest risk for both recurrent UTIs (rUTI) and vesicoureteral reflux (VUR), in order to allow for targeted voiding cystourethrogram (VCUG), while children at low risk could be observed [15]. The authors enrolled 500 subjects (305 RIVUR and 195 CUTIE) in their study. The mean age was 21 ± 19 months. In this study, 72 patients developed rUTI, out of which 53 also had VUR (10.6% of the total). The final model was developed with a set of variables including age, gender, race, weight, systolic blood pressure percentile, dysuria, urine albumin/creatinine ratio, prior antibiotics exposure, and current medication. Compared with children without rUTI-associated VUR, patients with rUTI-associated VUR were significantly more likely to be white (91% vs. 72%), taking over-the-counter or prescription medication (74% vs. 49), and have a higher index UTI temperature (mean 39.8 vs. 39.4 °C). The final model had an area under the curve of 0.761. The study concluded that the predictive model provides a promising performance to facilitate the individualized management of children with initial UTIs [15].

The research conducted by Ozkan et al. [16] aimed to identify if an AI model could predict the probability of cystitis and non-specific urethritis diseases with similar symptoms from the urinary tract and, if so, to identify which one performed the best. For this purpose, the results of routine examination, urinalysis, and diagnostic medical sonography of 59 patients were collected and composed as a UTI dataset. Four different artificial intelligence methods, i.e., decision tree (DT), SVM, random forest (RF), and ANN, which are widely used in medical diagnosis systems, were used to create classification structures. Accuracy, specificity, and sensitivity statistical measurements were used to determine the performance of the created models. The comparison of individual AI methods showed that ANN had the highest accuracy result of 98.3% for UTI diagnosis. Unlike clinical-based diagnosis, this ANN model only needs the variables of pollakiuria, suprapubic pain, and erythrocyturia to receive a proper diagnosis with similar accuracy. The conclusion of this study indicated that the possibility of making a decision about complicated UTIs using factors of suprapubic pain, pollakiuria, and urinalysis result, assisted by AI methods, is very much real and applicable in the modern world. It was shown that the ANN-based model structure could classify UTIs without the need for expensive laboratory tests and ultrasounds, and thus has a a lower diagnostic cost, shorter decision time, and no need for invasive methods. Additionally, different types of data augmentation can be used to increase the accuracy of the model.

The cohort study from 2019 performed by Gadalla et al. [17] was the first attempt to use cloudiness and immunological biomarkers in urine samples as key factors in machine learning algorithms (RF and SVM) for UTI prediction. The authors investigated whether it was possible to use clinical and urinary immunological biomarkers to predict UTIs. In their study, the researchers included female patients who presented in primary care with at least one of following symptoms: dysuria, urgency, or frequency. Patients with signs of complicated UTIs, current use of antibiotics, and functional or anatomical genitourinary tract abnormalities, as well as pregnant women, were excluded from further research. General practitioners (GPs) collected the information and evaluated the symptoms on a scale from 0 = no symptoms to 6 = severe to measure its intensity. In uncomplicated UTIs, white blood cells, red blood cells, epithelial cells, and microorganisms can cause the urine to become cloudy. Urine cloudiness was also reported by GPs following sample examination and emerged to be particularly helpful in ruling out uncomplicated UTI cases. During this study, 17 clinical and 42 immunological potential predictors for bacterial culture were found using RF or SVM coupled with recursive feature elimination. Urine cloudiness was the best performing clinical predictor to rule out (negative likelihood ratio [LR−] = 0.4) and rule in (LR+ = 2.6) UTIs. Using a more discriminatory scale to assess cloudiness (turbidity) further increased the accuracy of UTI prediction (LR+ = 4.4). Urinary levels of MMP9, NGAL, CXCL8, and IL-1β together had a higher LR+ (6.1) and similar LR−(0.4) compared with cloudiness. Clinical and urinary immunological biomarkers for UTI diagnosis are important predictors and could be used to develop a point-of-care test for UTIs, but require further validation.

Heckerling and colleagues [18] used ANN coupled with genetic algorithms to determine combinations of clinical variables optimized for predicting UTIs. The ANN examined 212 women enrolled in the study aged between 19 and 84 with symptoms of UTIs. Confirmation of infections in the urinary tract was defined based on different criteria in separate models, as uropathogen counts of ≥10^5^ colony-forming units (CFU) per milliliter and uropathogen counts of ≥10^2^ CFU per milliliter. Five-variable sets were created that classified cases of urinary tract infection and non-infection with receiver operating characteristic (ROC) curve areas that ranged from 0.853 (95% CI, 0.796–0.909) for uropathogen counts of ≥105 CFU per milliliter to 0.792 (95% CI, 0.726–0.858) for uropathogen counts of ≥102 CFU per milliliter. Network influence analyses revealed that some factors predicted urine infection in unexpected ways, and interacted with other variables when making predictions. While they found that cloudiness was associated with an increased LR+, their genetic algorithm did not retain it for the creation of the neural network. It is possible that this reflects the differences between neural networks and RF models [18].

**Table 1:** An overview of the current knowledge regarding various AI models in UTI diagnostics.

Authors	Cohort Size	Research Type	Top Performing Algorithm	Sensitivity (%)	Specificity (%)	Predictors Used in Developing of AI Models
Taylor et al. [13]	80.387	Retrospective cohort study	XGBoost	61,7 (60.0–63.3)	94.9 (94.5–95.3)	Age, gender, UA WBC (white blood cells), UA nitrates, UA leukocytes, UA bacteria, UA blood, UA epithelial cells, history of previous UTI, and dysuria
Burton et al. [14]	212.554	Retrospective cohort study	XGBoost (combined)	95.2 [+/−0.22]	60.93 [+/−0.62]	Demographics, historical urine culture results, and clinical details
Ozkan et al. [16]	59	Retrospective study	ANN	97.77	100	Pollacuria, suprapubic pain, and erythrocyturia
Advanced Analytics Group of Pediatric Urology et al. [15]	500 (children)	Observational cohort study	NA	NA	NA	Age, gender, race, weight, SBP (percentile), dysuria, ACR, and current and prior antibiotics
Gadalla et al. [17]	183	Retrospective cohort study	RF/SVM	NA	NA	Urine cloudness and urinary levels of MMP9, NGAL, CXC8, and IL-β
Heckerling et al. [18]	212	Retrospective cohort study	ANN + genetic algorithm	82.1 (69.2–90.7)	74.4 (66.6–80.9)	Urinary frequency; dysuria; foul urine odor; symptom duration; history of diabetes; leukocyte esterase on a urine dipstick; and red blood, cells, epithelial cells, and bacteria upon urinalysis

ACR—urine: albumin/creatine ratio; AI—artificial intelligence; ANN—artificial neutral networks; NA—not available; RF—random forest; SBP—systolic blood pressure; SVM—support vector machine; UA—urinalysis.

## 4. Conclusions and Future Directions

AI algorithms can reveal parsimonious variable sets that are accurate at predicting urinary tract infections, as well as novel relationships between symptoms, urinalysis findings, and inflammatory processes in the urinary tract. Accurate and rapid decision making can assist physicians in daily practice, especially considering children and infants. The literature examined here confirms the relevance of AI models in UTI diagnosis, whereas it has not yet been established which model is preferable for infection prediction in adult patients and in pediatric populations. The challenge is that a tremendous amount of big data are needed in order to construct a base for the application of a machine learning algorithm. Using new techniques in medicine could decrease the amount of time required for proper UTI diagnosis, which benefits from quick and proper treatment. Hopefully deep learning methods will prevent the overuse of anti-microbial drugs, which is particularly important in children. Artificial intelligence models have achieved a high performance in retrospective studies, but further studies are required in order to introduce advanced technology into everyday healthcare, nephrology, and urology, which could be beneficial, especially in children with recurrent urinary tract infections.

## References

[1] Stamm W.E., Norrby S.R. (2001). Urinary Tract Infections: Disease Panorama and Challenges. J. Infect. Dis..

[2] Flores-Mireles A.L., Walker J.N., Caparon M., Hultgren S.J. (2015). Urinary Tract Infections: Epidemiology, Mechanisms of Infection and Treatment Options, Nature Reviews. Microbiology.

[3] Wilson M.L., Gaido L. (2004). Laboratory Diagnosis of Urinary Tract Infections in Adult Patients. Clin. Infect. Dis..

[4] Schmiemann G., Kniehl E., Gebhardt K., Matejczyk M.M., Hummers-Pradier E. (2010). The Diagnosis of Urinary Tract Infection: A Systematic Review. Dtsch. Arztebl. Int..

[5] Choudhury A., Asan O. (2020). Role of artificial intelligence in patient safety outcomes: Systematic literature review. JMIR Med. Inform..

[6] Choudhury A., Renjilian E., Asan O. (2020). Use of machine learning in geriatric clinical care for chronic diseases: A systematic literature review. JAMIA Open.

[7] Secinaro S., Calandra D., Secinaro A., Muthurangu V., Biancone P. (2021). The role of artificial intelligence in healthcare: A structured literature review. BMC Med. Inform. Decis. Mak..

[8] McCarthy J. (2007). What Is Artificial Intelligence? Stanford University, Computer Science Department. http://www-formal.stanford.edu/jmc/whatisai/whatisai.

[9] FDA Proposed Regulatory Framework for Modifications to Artificial Intelligence/Machine Learning (AI/ML)-Based Software as a Medical Device (SaMD). https://www.fda.gov/files/medical%20devices/published/US-FDA-Artificial-Intelligence-and-Machine-Learning-Discussion-Paper.pdf.

[10] Asan O., Bayrak A.E., Choudhury A. (2020). Artificial intelligence and human trust in healthcare: Focus on clinicians. J. Med. Internet Res..

[11] FDA (2017). What Are Examples of Software as a Medical Device?. https://www.fda.gov/medical-devices/software-medical-device-samd/what-are-examples-software-medical-device.

[12] Xie G., Chen T., Li Y., Chen T., Li X., Liu Z. (2020). Artificial Intelligence in Nephrology: How Can Artificial Intelligence Augment Nephrologists’ Intelligence?. Kidney Dis..

[13] Taylor R.A., Moore C.L., Cheung K.H., Brandt C. (2018). Predicting urinary tract infections in the emergency department with machine learning. PLoS ONE.

[14] Burton R.J., Albur M., Eberl M., Cuff S.M. (2019). Using Artificial Intelligence to Reduce Diagnostic Workload without Compromising Detection of Urinary Tract Infections. BMC Med. Inform. Decis. Mak..

[15] Advanced Analytics Group of Pediatric Urology and ORC Personalized Medicine Group (2019). Targeted Workup after Initial Febrile Urinary Tract Infection: Using a Novel Machine Learning Model to Identify Children Most Likely to Benefit from Voiding Cystourethrogram. J. Urol..

[16] Ozkan I.A., Koklu M., Sert I.U. (2018). Diagnosis of Urinary Tract Infection Based on Artificial Intelligence Methods. Comput. Methods Programs Biomed..

[17] Gadalla A.A.H., Friberg I.M., Kift-Morgan A., Zhang J., Eberl M., Topley N., Weeks I., Cuff S., Wootton M., Gal M. (2019). Identification of clinical and urine biomarkers for uncomplicated urinary tract infection using machine learning algorithms. Sci. Rep..

[18] Heckerling P.S., Canaris G.J., Flach S.D., Tape T.G., Wigton R.S., Gerber B.S. (2007). Predictors of Urinary Tract Infection Based on Artificial Neural Networks and Genetic Algorithms. Int. J. Med. Inform..

